# Reduced Dietary Selenium Impairs Vascular Function by Increasing Oxidative Stress in Sprague-Dawley Rat Aortas

**DOI:** 10.3390/ijerph14060591

**Published:** 2017-06-02

**Authors:** Ana Stupin, Anita Cosic, Sanja Novak, Monika Vesel, Ivana Jukic, Brigita Popovic, Krunoslav Karalic, Zdenko Loncaric, Ines Drenjancevic

**Affiliations:** 1 Department of Physiology and Immunology, Faculty of Medicine, Josip Juraj Strossmayer University of Osijek, Cara Hadrijana 10E, HR-31000 Osijek, Croatia; cavka.ana@gmail.com (A.S.); anitaa3006@gmail.com (A.C.); sanjanov@gmail.com (S.N.); monika.vesel19@gmail.com (M.V.); grizelj.ivana@gmail.com (I.J.); 2 Department of Agroecology, Faculty of Agriculture, Josip Juraj Strossmayer University of Osijek, HR-31000 Osijek, Croatia; brigita.popovic@pfos.hr (B.P.); krunoslav.karalic@pfos.hr (K.K.); zdenko.loncaric@pfos.hr (Z.L.)

**Keywords:** selenium, vascular function, endothelium, oxidative stress, rats

## Abstract

This study aimed to determine whether low dietary Se content affects the function and mechanisms mediating the vascular relaxation of rat aortas, and to test the role of oxidative stress in observed differences. Male Sprague Dawley (SD) rats were maintained for 10 weeks on low Se (low-Se group; N = 20) or normal Se content (norm-Se group; N = 20) rat chow. Dose responses to acetylcholine (ACh; 10^−9^–10^−5^M) and the response to reduced pO_2_ were tested in noradrenaline-precontracted aortic rings in the absence/presence of the nitric oxide synthase (NOS) inhibitor nitro-l-arginine methyl ester (l-NAME), the cyclooxygenase 1 and 2 (COX-1, 2) inhibitor Indomethacin, and the antioxidative agent Tempol in tissue bath. mRNA expression of glutathione peroxidase 1 (GPx1), catalase (CAT), and Cu/Zn superoxide dismutase (SOD) was measured in rat aortas. Oxidative stress (Thiobarbituric Acid Reactive Substances; TBARS), antioxidative plasma capacity (ferric reducing ability of plasma assay; FRAP), and protein levels of GPx1 were measured in plasma and serum samples, respectively. Reduced ACh-induced relaxation (AChIR) (dominantly mediated by NO) in the low-Se group compared to the norm-Se group was restored by Tempol administration. Hypoxia-induced relaxation (HIR) (dominantly mediated by COX-1, 2), TBARS, and FRAP as well as GPx1 serum concentrations were similar between the groups. mRNA GPx1 expression in rat aortas was significantly decreased in the low-Se compared to the norm-Se group. These data suggest that low dietary Se content increases the local oxidative stress level, which subsequently affects the NO-mediated vascular response.

## 1. Introduction

It is well accepted that an increased oxidative stress level, which represents an imbalance between prooxidants and antioxidants in favor of prooxidants, is one of the main contributors to the development and progression of various pathological conditions linked to cardiovascular diseases (CVDs), including atherosclerosis, hypertension, diabetes mellitus, hypercholesterolemia, obesity, etc. [1,2]. Furthermore, endothelial dysfunction is considered to be a key early event in the development of CVDs [3,4]. Endothelial cells are particularly susceptible to oxidative stress, not only through reactive oxygen species (ROS)-mediated cell death, but also because of the compromised bioavailability of the normally protective mediator, NO [3,4]. Consequently, in the past few decades, coronary artery disease, stroke, peripheral vascular disease, hypertension, and heart failure are examples of CVDs that are considered to be valid targets for antioxidant therapy [5,6,7,8,9]. Antioxidant therapy includes a wide variety of different approaches—from dietary interventions to the intake of specific antioxidants. Still, the results of a number of studies on the benefits of antioxidants in vascular health have not been conclusive, and there is still no consensus about the most effective antioxidant therapeutic approach in CVDs [5,10,11,12].

Se has been recognized as a biological trace element which plays an important role in host oxidative defense by virtue of its ability to incorporate into antioxidant enzymes as selenocysteine (SeCys) [13,14]. Glutathione peroxidase (GPx) and thioredoxin reductase (TR) are the main representatives of Se-containing enzymes in humans [15]. Previous studies have shown that an increased activity of Se-dependent GPx might prevent ROS-induced protein and DNA damage, while TRs are named accordingly by their ability to reduce oxidized thioredoxin (Trx) [16]. Furthermore, a growing body of evidence indicates that endothelial selenoproteins are involved in the regulation of: (1) the vascular tone by maintaining the superoxide anion/NO balance; (2) cell adhesion by controlling cell adhesion molecule expression; (3) apoptosis via inhibition/activation of apoptosis signal-regulating kinase-1; and (4) eicosanoid production by controlling the activity of the cyclooxygenase 1 and 2 (COX-1, 2) and lipoxygenases [17,18]. Accordingly, it became evident that Se and Se-contained enzymes may be directly involved in the regulation of vascular inflammatory processes and atherogenesis [16]. Se dietary intake may influence the pathogenesis of various CVDs [19,20,21]. Recent studies have shown that the activities of GPx and TR can be altered by manipulating the Se content of a diet [18,21,22,23,24,25]. Furthermore, Se dietary deficiency has been related to the etiology of various CVDs, while its supplementation contributed to the protection of human endothelial cells from oxidative injury [26]. Thus, it became evident that dietary Se deficiency, which occurs due to its inadequate intake, decreased absorption, or increased loss, can be an important factor in the promotion and progression of CVDs, while on the other hand, Se supplementation may prevent them. According to the World Health Organization, a diet containing 0.1 mg Se/kg of food is enough for normal growth and reproduction in mammals [27]. When taking into account that the difference in Se concentrations in food sources depends on its content in soil, and that the geographical distribution of Se ranges from Se deficient regions to Se rich regions [28], Se deficiency may present a health issue in populations living in regions with low Se content in soil.

Even though Se was proposed to have an atheroprotective function, little is known about the actual requirement and function of Se in maintaining normal vascular and endothelial function. Thus, the aim of this study was: (1) to determine whether low dietary Se content affects vascular relaxation mechanisms in rat aortic ring preparation; (2) to test the role of oxidative stress and antioxidative enzymes in observed differences; and (3) to elucidate the mechanisms mediating vascular relaxation during dietary Se level modulation. The main hypothesis is that low dietary Se content reduces relaxation of the aortic rings in Sprague-Dawley (SD) male rats, and that increased local oxidative stress plays an important role in this impairment. Under this hypothesis, exogenous Tempol, which is considered a general purpose redox cycling agent rather than a specific superoxide dismutase (SOD)-mimetic compound, would be expected to reverse any impairment of vascular reactivity induced by low dietary Se content. mRNA expression of important antioxidant enzymes in rat aortas is expected to be decreased in rats fed with low Se content chow.

## 2. Materials and Methods

### 2.1. Experimental Animals

The animals were grown and housed at the animal care facility of the Faculty of Medicine at the Josip Juraj Strossmayer University of Osijek, Croatia, which is a registered and certified user/breeder of mice and rats for educational and scientific purposes. The procuration of animals, the husbandry, and all experimental procedures conformed to the ‘European Convention for the Protection of Vertebrate Animals used for Experimental and other Scientific Purposes’ (Council of Europe No 123, Strasbourg 1985). Experiments were approved by the Ethical Committee of the Faculty of Medicine, University of Osijek (Class: 602-04/14-08/06, No: 2158-61-07-14-05), and authorized by the Ministry of Agriculture of the Republic of Croatia (Class: UP/I-322-01/14-01/90, No: 525-10/0255-15-4).

A total of forty, 4-week-old male SD rats were fed with two types of custom-made rat chow for 10 weeks. The animals were randomly divided into two groups according to the type of custom-made rat chow administered. The low-Se group was fed with the plain rat chow with low Se content (0.030 mg/kg) (N = 20). The norm-Se group was fed with the rat chow with normal Se content (0.363 mg/kg) (N = 20). Fourteen rats from each group were used for functional aortic ring reactivity experiments, and six animals from each group were used for measurement of Se content and molecular experiments in thoracic aorta tissue.

According to the American Institute of Nutrition (AIN) and the TestDiet^®^ AIN-93 Growth Purified Diet, which represents the recommended growth diet for rodents, optimum Se content is 0.24 mg Se/kg of food [29]. In accordance with AIN-93, the low Se rat chow used in this study contained 0.030 mg Se/kg of food, and the normal Se rat chow contained 0.363 mg Se/kg of food. Custom made rat chow was prepared at the Faculty of Agriculture of the University of Osijek, Croatia, based on the recipe of Mucedola (Mucedola S.R.L., 20019 Settimo Milanese, MI, Italy) (composition: wheat, maize, toasted soya extraction flour, gluten maize flour, wheat straw, fish flour, medical herb flour, dicalcium phosphate, calcium carbonate from calcite calcine, sodium chloride, whey powder, soybean oil, yeast; additives (per kg): vitamin A 14,400 IU, vitamin D3 1260 IU, Fe 180 mg, Mn 54 mg, Zn 67.5 mg, I 0.90 mg, Co 0.63 mg; technological additives: sepiolite 880 mg, humidity 12.00%, raw protein 18.50%, oil and row grass 3.00%, raw fiber 6.00%, rough ash 7.00%). The ingredients for the rat chow preparation were standard except the Divana wheat, which was plain but low in Se, or wheat that was bio fortified to the normal Se content (according to the Components of the AIN-93 Diets) [29]. The methods and amounts of applied Se were in accordance with earlier published results and with agronomic biofortification strategies in order to improve nutrition [30,31].

SD rats were housed doubly in shoebox-style cages with free access to rat chow and tap water, and maintained on a 12:12 h light:dark cycle. Rat weight was measured weekly from the 4th until the 14th week of age.

### 2.2. Surgery, Blood Collection, and Aortic Ring Acquisition

At the age of 14 weeks, the aortic ring experiments were conducted. For that purpose, the rats were anaesthetized with a combination of ketamine (75 mg/kg) and midazolam (2.5 mg/kg). After decapitation, the descending thoracic aorta was carefully and promptly dissected from the connective tissue, placed in an oxygenated modified Krebs-Henseleit solution, and cut into rings of about 2–3 mm in length. From one thoracic aorta, four aortic rings were obtained for functional vascular experiments (n—number of aortic rings). The general procedures for thoracic aortic ring acquisition and preparation were done according to the protocol already described in our laboratory. One part of the isolated thoracic aortas was stored in liquid nitrogen until the measurement of Se content in the aorta tissue (six rats from each experimental group).

### 2.3. Measurement of Isometric Tension of Rat Aortic Rings and Assessment of Aortic Ring Reactivity to Acetylcholine and Reduced pO_2_

The aortic ring experiments were done according to the protocol already described in our laboratory [32]. Short segments from each end of the isolated aorta were severed and discarded, whereas the rest of the vessel was cut into rings (of about 3–4 mm in length). These rings were mounted in tissue bath chambers containing the Krebs-Henseleit solution (maintained at 37 °C) with a 95% O_2_/5% CO_2_ compressed gas mixture bubbling through and connected to pressure transducers as part of an Experimetria vessel ring preparation setup (purchased from Experimetria Ltd., Budapest, Hungary). The data were continuously documented on a computer and later analyzed. The passive tension for each ring was set at 2.0 g. The vessels were allowed to equilibrate and stabilize for 1 h, replacing the Krebs-Henseleit solution every 15 min with fresh solution and readjusting passive tension to 2.0 g as needed. Subsequently, the intactness of endothelium was tested by precontracting the rings with 10^−7^ M (final concentration) noradrenaline (NA), allowing stabilization for 5 min and inducing relaxation with 10^−5^ M acetylcholine (ACh). If the vessel ring failed to relax, it was not used for further studies. If the vessel ring relaxed, it was washed three times with fresh solution and allowed to equilibrate for 30 min, with washing occurring at 10 min intervals. After the initial test for vessel viability and endothelial integrity, the rings were stabilized and then maximal contraction was induced with 60 mM KCl+10^−7^ M NA. When a plateau was reached, the rings were washed three times with fresh solution and allowed to equilibrate for 30 min, with washing occurring at 10 min intervals. 

After this phase in the ACh relaxation protocol, aortic rings were precontracted with 10^−7^ M NA for 5 min, and cumulative concentration-response curves to ACh were created by increasing the ACh concentration in the tissue bath by the successive addition of appropriate dilutions of stock solutions to achieve final bath concentrations of 10^−9^ to 10^−5^ M ACh. ACh relaxation protocol was done in the absence and in the presence of one of the inhibitors or scavengers: (1) the nitric oxide synthase (NOS) inhibitor, nitro-l-arginine methyl ester (l-NAME, 3 × 10^−4^ M); (2) the COX-1, -2 inhibitor, Indomethacin (10^−5^ M); and (3) the superoxide scavenger, Tempol (10^−5^ M) in tissue bath. The relaxation was expressed as the percentage of remaining contraction of the NA-induced vasoconstriction.

To test the vascular response to reduced pO_2_, the rings were precontracted with NA at a final concentration of 10^−7^ M and allowed to stabilize at a maximum response (≅5 min). Then the gas equilibration mixture in the tissue bath was changed from 95% O2/5% CO2 to mixtures containing 0% O_2_/5% CO_2_/95%N_2_. Since the volume of each tissue bath was small (10 mL), equilibration with the gases required only a few minutes. To verify that the ring was viable at the end of the hypoxic relaxation protocol, the bath was changed to 95% O_2_/5% CO_2_ for 20 min. If the aortic ring did not contract and develop a force approximately equal to the initial force development in response to 95% O_2_, the force values obtained during exposure to hypoxia were eliminated from the analysis [33]. The hypoxic relaxation protocol was repeated with the presence of one of the inhibitors or scavengers: l-NAME, Indomethacin, and Tempol.

### 2.4. Measurement of Se Content in Whole Blood and in Thoracic Aorta Tissue

Previous studies have shown that intracellular concentration of biological trace elements, such as the concentration of trace elements in lymphocyte, is a better and more sensitive indicator of its status than plasma concentration [34]. Thus, Se content in whole blood was measured, as well as its content in thoracic aorta tissue. All tissue samples for measuring Se concentration were digested with 10 mL of a 5:1 mixture of HNO_3_ and H_2_O_2_ at 180 °C for 60 min in a microwave oven (CEM Mars 6). The solution of digested samples was prepared for measuring Se concentration by adding 5 mL of concentrated HCl in order to reduce Se^6+^ to Se^4+^. The concentrations of Se in solutions of digested tissue samples were determined by inductively coupled plasma optical emission spectrometry (ICP-OES) (Optima 2100 DV, PerkinElmer Inc., Waltham, MA, SAD). Each batch of tissue samples run on the ICP was analyzed with an internal pooled plasma control and with the reference material (Chicken, NCS Certified Reference Material—NCS ZC73016, China National Analysis Center) prepared in the same way as the other tissue samples. All samples were analyzed in duplicate.

### 2.5. Measurement of Oxidative Stress and Antioxidant Capacity

Blood samples were collected from the decapitation site, centrifuged at 3500 rpm for 10 min, and serum samples were stored at −80 °C until the experiment. Experiments were performed according to the protocol that was already described in our laboratory [35]. As a direct indicator of oxidative stress, the spectrophotometric Thiobarbituric Acid Reactive Substances (TBARS) method was used for measuring the products of lipid peroxidation with malondialdehyde (MDA) as standard (μmol l−1 MDA). The products bind to a thiobarbituric acid (TBA) at low pH. Since the method is non-specific because the other substances bind to a TBA (including proteins), trichloroacetic acid (TCA) is first added to the sample to precipitate the proteins, and after that the supernatant was used for the measurements [36]. The absorbance of the sample was measured at 572 and 532 nm.

Antioxidant capacity was assessed using the ferric reducing ability of plasma assay (FRAP). Fe^3^+-TPTZ (2,4,6-tris(2-pyridyl)-s-triazine) is reduced to Fe^2^+-TPTZ in the presence of antioxidants, and blue discoloration occurs. The absorbance of the sample was measured at 593 nm (Nanophotometer P300 UV/VIS, IMPLEN), using Trolox as a standard (mmol Trolox) [37].

### 2.6. Measurement of Glutathione Peroxidase 1 Serum Concentration

The serum concentration of glutathione peroxidase 1 (GPX1) was measured with an enzyme-linked Immunosorbent Assay (ELISA) Kit (Uscn Life Science Inc. Wuhan., P.R. China, Cat. No. E90295Ra). The detection range of the ELISA kit was 1.56–100 ng/mL. The minimum detectable dose of rat GPX1 is typically less than 0.65 ng/mL. Intra-Assay Precision (precision within an assay): coefficient of variation (CV) < 10% and Inter-Assay Precision (precision between assays): CV < 12%.

### 2.7. mRNA Expression of Antioxidative Enzymes in Rat Aortas

Aortas for molecular study were isolated and collected for the determination of gene expression using quantitative polymerase chain reaction (rtPCR). Samples were rapidly frozen in liquid nitrogen and stored at −80 °C until the experiment. Homogenization of samples and total RNA was extracted using One Step RNA Reagent (Bio Basic Canada Inc., Markham, ON, Canada), according to the protocol in [38]. RNA concentration and purity was assessed using Nanophotometer P300 UV/VIS, IMPLEN, and RNA integrity was checked on 1% agarose gel. Purification of samples and the obtaining of cDNA was made according to the manufacturer: Sigma-Aldrich, St. Louis, MO, USA) and Applied Biosystems, Foster City, CA, USA.

To determine the expression of mRNA for antioxidative enzymes (GPx1, H_2_O_2_ scavenger—CAT and/or Cu/Zn SOD), quantitative real-time PCR was performed on a CFX96 system (Bio Rad, Hercules, CA, USA), and their relative expression was normalized to the expression of housekeeping gene 18S rRNAgene.

### 2.8. Reagents

NA, ACh, l-NAME, Indomethacin and Tempol were purchased from Sigma-Aldrich. Ketamine and midazolam were obtained from Pfizer(New York City, NY, SAD). The Krebs-Henseleit solution (composition: 113 mM NaCl, 4.7 mM KCl, 1.2 mM MgSO_4_ × 7H_2_O, 22 mM NaHCO_3_, 1.2 mM KH_2_PO_4_, 11 mM glucose, 2.5 mM CaCl_2_ × 2H_2_O, 0.026 mM ethylenediaminetetraacetic acid (EDTA); pH 7.4) was prepared from EDTA and purchased from Sigma-Aldrich, CaCl_2_ × 2H_2_O and NaHCO_3_ from Merck KGaA, with the rest of the chemicals purchased from Kemika, Zagreb, Croatia. Gas mixtures were purchased from Messer, Zagreb, Croatia. Total RNA was extracted using ONE STEP RNA Reagent (Bio Basic Canada Inc., Markham, ON, Canada) according to the manufacturer’s protocol. Deoxyribonuclease I kit (Sigma-Aldrich) was used to purify RNA. Complementary DNA (cDNA) was synthetized by a High Capacity cDNA kit. Synthetized cDNA was diluted in nuclease free water (Sigma Aldrich, Germany) and used for real-time PCR. The chemicals used to determine the oxidative stress were thiobarbituric acid (TBA; Sigma-Aldrich), trichloroacetic acid (TCA; PanReac, Barcelona, Spain), and 1,1,3,3-tetramethoxypropane (TMP; Sigma-Aldrich), and for FRAP analysis Trolox (Sigma-Aldrich), 2,4,6-tris(2-pyridyl)-s-triazine (TPTZ; Sigma-Aldrich), iron (III) chloride hexahydrate (FeCl3·6H_2_O; Sigma-Aldrich), and sodium acetate trihydrate (Kemika, Zagreb, Croatia).

### 2.9. Statistical Analysis

Data were obtained by software provided by the setup manufacturer (Experimetria Ltd.) and then transferred into a spreadsheet for statistical analyses with SigmaPlot v11.2 (Systat Software, Chicago, IL, USA). All data are summarized as means ± SEM. Two-way ANOVA tests and Bonferroni post hoc tests were used to test differences in ACh-induced relaxation among groups. Half maximal effective concentration (LogEC50) ACh values were compared by student *t*-tests or one-way Analysis of Variance (ANOVA) tests followed by a Holm-Sidak pairwise multiple comparison when appropriate. A student *t*-test was used to test the difference in hypoxia-induced dilation and antioxidative gene expression among groups. A probability of *p* ≤ 0.05 was considered to be statistically significant. GraphPad Prism v5.0 (GraphPad Software, Inc., La Jolla, CA, USA) was used for the graphic presentation of the obtained results.

## 3. Results

### 3.1. Body Weight of Experimental Animals

**Figure 1:**
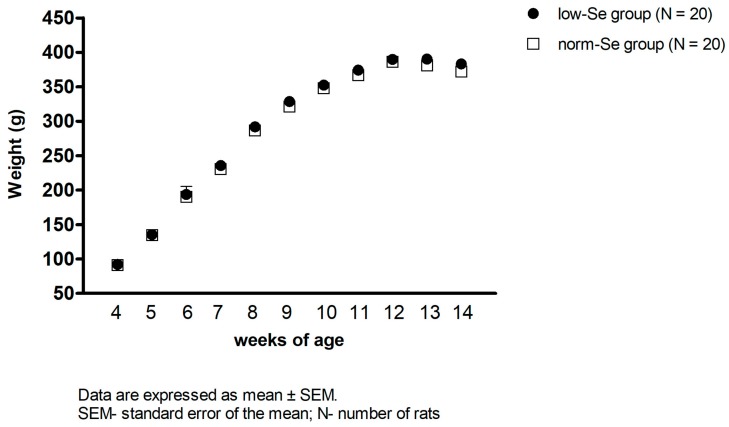
Body weight of experimental animals during dietary Se manipulation (from the 4th until the 14th week of age). There was no significant difference in the experimental animals’ body weight between the low-Se and the norm-Se groups of rats (*p* > 0.05).

Weekly rat weight measurement from the 4th until the 14th week of age has demonstrated that there was no difference in the animals’ body weight between the two experimental groups of rats during the whole diet protocol (Figure 1).

### 3.2. Acetylcholine Induced Relaxation of Isolated Rat Aortic Rings

**Figure 2:**
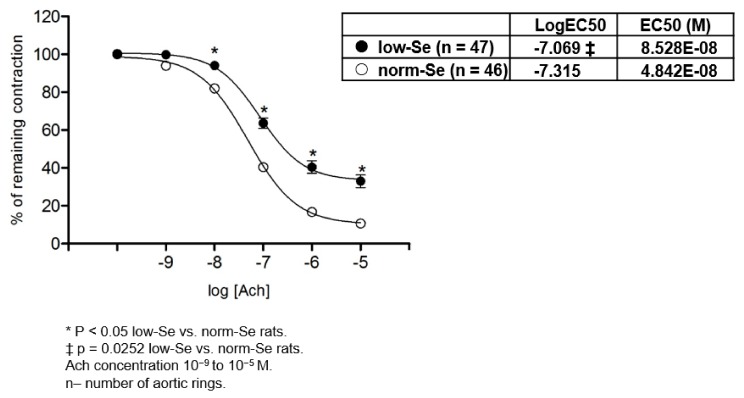
ACh induced relaxation (AChIR) of isolated rat aorta rings in the low-Se and norm-Se groups. AChIR was significantly impaired in the low-Se group when compared to the norm-Se group of rats with a 10^−8^–10^−5^ M ACh concentration. Norm-Se rats exhibited higher sensitivity to ACh compared to Low-Se rats (table). Half maximal effective concentration (EC50) presents concentration of ACh (M) which induces a response halfway between the baseline and maximum. LogEC50 values (shown in corresponding tables) were compared by a Student *t*-test.

The low-Se group of rats exhibited significantly reduced relaxation of isolated rat aortic rings to ACh compared to the norm-Se group of rats (Figure 2). Furthermore, analysis of isolated rat aortic ring sensitivity to ACh demonstrated that the low-Se rats exhibited reduced sensitivity of isolated aortic rings to ACh compared to isolated aortic rings of the norm-Se rats (table within Figure 2). Taken together, the 10-week feeding regime with low Se content rat chow resulted in impaired vasorelaxation of isolated rat aortic rings to ACh, as well as reduced sensitivity to ACh.

**Figure 3:**
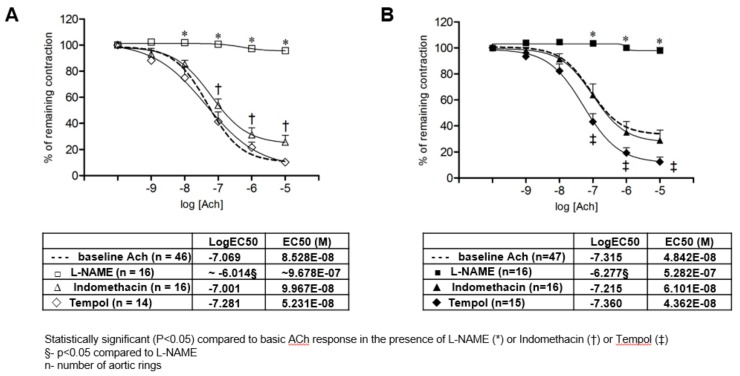
Mechanisms of AchIR response of isolated rat aorta rings in norm-Se (**A**) and low-Se rats (**B**). Used concentrations: ACh 10^−9^ to 10^−5^ M, l-NAME 3 × 10^−4^ M, Indomethacin 10^−5^ M, and Tempol 10^−5^ M. Half maximal effective concentration (EC50) presents the concentration of ACh (M) which induces a response halfway between the baseline and maximum.

The mechanisms mediating the AChIR response of isolated rat aortic rings in experimental groups of rats are presented in Figure 3. NO-mediated dilation of aortic rings was tested by the addition of the eNOS inhibitor l-NAME, and the cyclooxygenase-dependent pathway was tested by the addition of the non-selective COX-1, -2 inhibitor Indomethacin to the tissue bath. The presence of a high oxidative stress level was functionally assessed by the addition of Tempol, which is considered a multifunctional antioxidant rather than just a superoxide scavenger, to the tissue bath. In the norm-Se group, the presence of l-NAME and Indomethacin significantly reduced the AChIR of isolated rat aortic rings, which means that AChIR in norm-Se rats was mediated mainly by NO, with a significant contribution of COX-1, -2 vasodilator metabolites as well (Figure 3A). Tempol administration did not have any significant effect on AChIR in the norm-Se group of rats (Figure 3A), suggesting the oxidative stress level in the norm-Se group of rats was not increased. On the other hand, in the low-Se group, the presence of l-NAME, but not Indomethacin, significantly reduced the AChIR of isolated rat aortic rings, suggesting that AChIR was mediated mainly by NO without the contribution of COX-1, -2 vasodilator metabolites in the low-Se group of rats (Figure 3B). Importantly, Tempol administration restored the AChIR response in the low-Se group of rats to levels similar to the norm-Se group (Figure 3A), suggesting that reduced AChIR in low-Se rats is probably provoked by an increased oxidative stress level. Analysis of aortic ring sensitivity to ACh demonstrated that sensitivity to ACh in the presence of l-NAME was significantly decreased compared to the basic response or response to ACh in the presence of Indomethacin or Tempol in both the low-Se and norm-Se groups of rats (table within Figure 3A,B).

Figure 3A,B present the relaxation of isolated aortic rings to ACh (presented as log [ACh] of ACh concentration (10^−9^ to 10^−5^ M)) in norm-Se (Figure 3A) and low-Se groups of rats (Figure 3B). The presence of l-NAME and Indomethacin significantly reduced the AChIR of isolated rat aortic rings in the norm-Se group (Figure 3A), and only the presence of l-NAME significantly reduced AChIR in the low-Se group of rats (Figure 3B). Tempol administration did not have any significant effect on AChIR in the norm-Se group of rats (Figure 3A), while its administration significantly increased AChIR response in the low-Se group of rats (Figure 3B). Data were compared by two-way ANOVA and Bonferroni post hoc tests. Sensitivity to ACh in the presence of l-NAME was significantly decreased compared to the basic response or response to ACh in the presence of Indomethacin or Tempol in both the low-Se and norm-Se groups of rats. LogEC50 values were compared by one-way ANOVA followed by a Holm-Sidak pairwise multiple comparison.

There was no difference in NO, and COX-1, -2 vasodilator mediators contributed vasorelaxation to ACh in the low-Se and norm-Se groups of rats (l-NAME Log EC50 low-Se vs. norm-Se −6.014 vs. −6.277, *p* > 0.05; Indomethacin Log EC50 low-Se vs. norm-Se −7.001 vs. −7.215, *p* > 0.05). Tempol did not contribute differently to ACh vasorelaxation in low-Se or norm-Se rats (Tempol Log EC50 low-Se vs. norm-Se −7.281 vs. −7.360, *p* > 0.05). Obtained data were compared by two-way ANOVA and Bonferroni post hoc tests.

### 3.3. Hypoxia Induced Relaxation of Isolated Rat Aortic Rings

**Figure 4:**
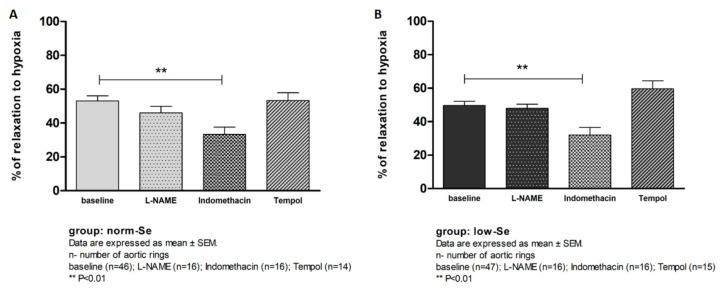
The mechanisms of hypoxia induced relaxation (HIR) response of isolated rat aorta rings in norm-Se (**A**) and low-Se group (**B**).

There was no statistically significant difference in the Hypoxia Induced Relaxation (HIR) of isolated rat aortic rings between the low-Se and norm-Se groups of rats (% of relaxation to hypoxia low-Se vs. norm-Se 49.63 ± 18.15 vs. 53.1 ± 21.16, *p* = 0.352). The mechanisms mediating HIR response in the experimental groups of rats were tested in the same manner as in the AChIR protocol (addition of l-NAME, Indomethacin, or Tempol in the tissue bath) and are presented in Figure 4**.** In both the low-Se and norm-Se groups, the presence of Indomethacin but not l-NAME significantly reduced HIR, suggesting that a vasodilator response to hypoxia was mediated mainly by COX-1, -2 vasodilator metabolites, with no significant contribution of NO in both the norm-Se and low-Se groups of rats (Figure 4A,B). Tempol administration did not have any significant effect on HIR response in either experimental group (Figure 4A,B).

In both the norm-Se and low-Se groups, the presence of Indomethacin significantly reduced the HIR of isolated rat aortic rings (Figure 4A,B). l-NAME and Tempol administration did not have any significant effect on HIR in either group of rats (Figure 4A,B).

### 3.4. Se Content in Whole Blood and in Thoracic Aorta Tissue

The measurement of Se content in whole blood and in thoracic aorta tissue demonstrated that Se content in both whole blood and in thoracic aorta tissue was significantly decreased in the low-Se group compared to the normal-Se group of rats (Table 1). These results confirmed that the experimental protocol was appropriately designed and conducted consistently.

### 3.5. Plasma Oxidative Stress (TBARS) and Antioxidant Capacity (FRAP)

The spectrophotometric measurement of the plasma oxidative stress level and antioxidant capacity has demonstrated that there was no significant difference in both plasma oxidative stress (TBARS µmol MDA low-Se 10.57 ± 0.04 vs. norm-Se 10.66 ± 0.12, *p* = 0.095) and antioxidant capacity levels (FRAP mmol Trolox low-Se 0.028 ± 0.003 vs. norm-Se 0.028 ± 0.01, *p* = 0.895) between the experimental groups of rats.

### 3.6. Glutathione Peroxidase 1 Serum Concentration

The measurement of GPX1 serum concentration using the enzyme-linked immunosorbent assay (ELISA) (demonstrated that there was no significant difference in the GPX1 serum concentration between the low-Se and norm-Se groups of rats (GPX1 ng/mL low-Se group 5.17 ± 1.05 vs. norm-Se group 4.85 ± 0.86, *p* = 0.516).

### 3.7. mRNA Expression of Antioxidative Enzymes in Rat Aorta

The determination of relative gene expression using quantitative rtPCR has demonstrated that mRNA expression of GPx1 in rat aortic tissue was significantly reduced in the low-Se group compared to the norm-Se group of rats (*p* = 0.017). CAT and Cu/Zn SOD mRNA expression in rat aorta did not differ between the two experimental groups of rats (Table 2).

## 4. Discussion

Many observational studies have shown that increased dietary intake or the high blood concentration of antioxidants are associated with a reduced risk of CVDs [5,6,7,8,9]. However, in order to understand at least one part of the mechanisms linking antioxidants to CVDs, it was necessary to assess the potential effects of antioxidant deficiency and its supplementation (e.g., nutrients biofortified with the Se, which are part of important antioxidant enzymes) on vascular function.

The salient finding of the present study is that low dietary Se content affected vascular relaxation mechanisms and attenuated the AChIR of rat aortic rings potentially due to increased oxidative stress in rat aortas (i.e., decreased mRNA expression of vascular GPx1). In vitro Tempol administration restored AChIR in rats fed with low Se chow, confirming an impaired function of the antioxidative defense system in low-Se rats. Furthermore, since AChIR (predominantly mediated by NO) was more affected by low-Se dietary content than HIR (predominantly mediated by COX-1, -2 metabolites), it seems that increased oxidative stress by low dietary Se primarily affected a NO-mediated response of rat aortic rings.

### 4.1. Se Dietary Content and Supplementation

As already mentioned, the Se dietary content in rat chow for both experimental groups was appropriate, since 0.030 mg Se/kg of food was considered low and 0.363 mg Se/kg of food was considered normal content in accordance with AIN-93 [29]. The biofortification of wheat in rat chow did not have any adverse effect on the weight of the rats in the different experimental groups (Figure 1), which is consistent with previous studies [17]. The feeding regimen used in these experiments was successful in producing normalized Se content in whole blood and in the thoracic aorta wall in the norm-Se group, compared to the low-Se group (Table 1).

### 4.2. Influence of Se Dietary Content on Vascular Function

Se oral supplementation in the form of a pill is widespread, and is available as an over-the-counter drug to the general population. It is well accepted that oxidative stress is one of the main contributors to endothelial dysfunction, and since Se is recognized for its proposed atheroprotective function and its antioxidant and anti-inflammatory properties, it is surprising that there is still a paucity of inconsistent data on the vascular effects of dietary Se modulation in both experimental animal and human studies [17,18]. For example, a sub-study of the SUpplementation en VItamines et Minéraux AntioXydants (SU.VI.MAX) study has shown no marked beneficial effects of long-term daily low-dose supplementation of antioxidant vitamins (vitamin C and E, beta carotene) and minerals (Se) on carotid structure and arterial stiffness in healthy middle-aged volunteers [5]. On the other hand, the long-term intake of Se, calcium, and dairy products were positively associated with capillary recruitment in skin nutritive microcirculation in healthy young men [39]. Furthermore, Se deficiency was associated with adverse arterial function (pulse wave velocity) in patients with a high risk for vascular events [40]. Even less is known about the biochemical processes and possible vascular mechanism changes affected by Se intake. 

In 1987, Funk et al. showed that Se deficiency can affect the formation of prostacyclin and other oxygenated metabolites of polysaturated fatty acids by rat and rabbit aorta, possibly by increasing lipid peroxidation [41]. Furthermore, Se deficiency, by decreasing GPx activity, makes human umbilical vein endothelial cells (HUVECs) susceptible to peroxide-induced inhibition of the COX activity of prostaglandin H_2_ synthase, resulting in decreased prostacyclin production [42], which is consistent with the results of the present study. Low Se intake leads to severe changes in the vessel walls in spontaneously hypertensive rats, whereas Se supplementation slows down the elastin degradation and degenerative changes of the vessel walls [17]. Se supplementation improved the impairment of endothelium-dependent vasorelaxation in thoracic aorta of diabetic rats, possibly by regulating the antioxidant enzyme and NO release [43]. Furthermore, ACh dependent relaxation of aortic rings has been shown to be significantly diminished in vitamin E- and Se-deficient rats with hypercholesterolemia, compared to vitamin E- and Se-sufficient rats with hypercholesterolemia [44]. Interestingly, 3 days of Se supplementation in the form of an intraperitoneal injection of sodium selenite enhanced ACh-induced endothelium-dependent relaxation of rat aortic rings compared to control rats [45]. To our knowledge, the present study is the first study investigating the influence of dietary Se modulation by biofortified wheat on vascular reactivity (to different stimuli, e.g., ACh, hypoxia) in healthy SD male rats, which confirmed the hypothesis that low Se dietary content impairs reactivity of rat aortas probably due to increased levels of oxidative stress.

### 4.3. Influence of Se Dietary Content on Oxidative Stress

The results of the present study have demonstrated that locally increased oxidative stress plays an important role in the impaired vascular relaxation responses in low-Se rats (Figure 3B, Table 2). Importantly, increased oxidative stress was found only locally in the blood vessel tissue and not in the serum of low-Se experimental rats (Table 2). In order to evaluate the local antioxidant properties of Se dietary content, we measured the mRNA expression of three antioxidative enzymes: GPx1, CAT, and Cu/Zn SOD in aortic tissue (Table 2). It was previously reported that these three enzymes act in a cooperative or synergistic way to ensure a global cell protection [46]. Due to the results of earlier studies in animal models which reported that GPx1 has a major role in the prevention of oxidative stress, it was suggested that GPx1 may also be important in the regulation of vascular and endothelial functions [47]. It has been demonstrated that mice that are heterozygous for GPx1 deficiency have endothelial dysfunction combined with structural vascular abnormalities [48]. Regarding potential mechanisms mediating the influence of GPx1 deficiency and endothelial dysfunction, it has been shown that GPx1 deficiency apparently decreases bioavailable NO in mice [49]. Se dietary deficiency has been shown to redistribute intracellular Se among the selenoproteins and GPx proteins [50]. Since GPx1 has been shown to be highly sensitive, and GPx2 and GPx4 more resistant to changes in Se dietary content, there has been much interest in the effects of dietary Se modulation on GPx activity (especially red blood cell GPx) and mRNA levels, since these may provide potential biomarkers of Se status [51,52,53,54]. Accordingly, studies on experimental animal models tended to demonstrate this potentially clear relationship between Se content with the activity of its corresponding enzyme (e.g., GPx, TR). Wu et al. showed a positive relationship between dietary Se and the antioxidant capacity of rat arterial walls [26]. They reported that the regulation mechanisms of transcriptional TR and GPx genes by Se in rat arterial walls are different: regulation of the expression of TR was mediated by ROS, but expression of GPx was influenced by Se status in arterial walls [26]. Se supplementation in a form of selenomethionine significantly increased the GPx1 activity in the whole blood of spontaneously hypertensive rats compared to the rats on an adequate Se diet [18]. Se supplementation improved the redox status and increased antioxidant levels in poultry as well [55]. The increase of GPx1 (serum activity and/or mRNA expression in liver) due to dietary Se supplementation has been found in rodents [56,57], and GPx activity in different tissues has been shown to be affected by Se availability to differing extents [58]. Our results are consistent with these findings, since we demonstrated a significantly higher mRNA expression of GPx1 in aorta walls in rats that were fed with Se biofortified rat chow relative to normal Se values, compared to the low-Se rat group (Table 2). While GPx1 is a Se-dependent peroxidase, CAT is considered a Se-independent redox-protective peroxidase that is functionally linked to the selenoenzymes of the thioredoxin reductase family through their thioredoxin cofactors [59]. Thus, Se dietary intake may have impact (direct and indirect) on both GPx1 and CAT. Consistent with these data, the results of the present study have demonstrated decreased GPx1 mRNA expression in rat aortas in Se deficient rats compared to the rats fed by Se biofortified rat chow. In addition, while most studies were focused on the effects of Se deficiency/supplementation on different forms of GPx activities, and others have investigated the effects of Se supplementation on GPx activity in different tissues [24,60], there is a paucity of functional data on the influence of a changed oxidative milieu due to dietary Se modulation on vascular function. Besides its superoxide dismutase mimetic action, Tempol has the efficacy to metabolize cellular O_2_ and H_2_O_2_, as well as to protect cells from the damaging effects of the hydroxyl radical (·OH). Thus, Tempol is considered a multifunctional antioxidant that reacts with a diverse range of biological oxidants, and it serves as an appropriate substance for indirect general oxidative stress assessment in functional vascular experiments [61]. The present study is the first to demonstrate that Tempol administration restored vascular relaxation to ACh in rats that were fed low Se content rat chow, suggesting that AChIR attenuation in those rats could occur to increase oxidative stress (Figure 3B).

This study had limitations that should be noted. Since the salient finding of the present study is that low dietary Se content affected vascular relaxation of rat aortic rings due to locally increased oxidative stress in rat aortas, the lack of direct measurement of oxidative stress in aortic tissue (e.g., the visualization of emerging ROS in aortic tissue) could be considered a study limitation. Such measurements should be taken into account in future studies evaluating the interrelation between Se dietary modulation, oxidative stress level, and vascular function.

## 5. Conclusions

In summary, this is the first functional vascular study that: (1) confirms the deleterious effect of low dietary Se content on macrovascular function in SD rats; (2) demonstrates a significant effect of increased oxidative stress on AChIR attenuation in low-Se rats; (3) indicates that a possible cause of increased oxidative stress in low-Se rats is the decreased mRNA expression of an important antioxidant enzyme, GPx1; (4) shows that the dietary Se supplementation to normal Se levels by natural biofortification of wheat improves the AChIR of rat aortas; and (5) indicates that since AChIR (predominantly mediated by NO) was more affected by low dietary Se content than HIR (predominantly mediated by COX-1, -2 metabolites), that increased oxidative stress affects a NO-mediated response probably due to the decreased bioavailability of NO in rat aortas.

## Figures and Tables

**Table 1:** Se content in whole blood and in thoracic aorta tissue.

Experimental Group		Low-Se Group	Norm-Se Group
**Whole blood**	Se (μg/mL)	0.45 ± 0.01	0.54 ± 0.02 ***
**Thoracic aorta tissue**	Se (μg/mg)	0.12 ± 0.01	0.20 ± 0.01 ***

Data are expressed as mean ± SEM. SEM- standard error of the mean. *** *p* < 0.001 low-Se vs. norm-Se group.

**Table 2:** Relative mRNA expression of GPx1, CAT and Cu/Zn SOD genes in thoracic aorta tissue.

Experimental Group	GPx1	CAT	Cu/Zn SOD
**low-Se group**	1.70 ± 0.37 *	8.90 ± 0.84	1.91 ± 0.24
**norm-Se group**	3.52 ± 0.37	15.64 ± 3.19	2.13 ± 0.37

Data are presented as mean ± SEM. GPx1—glutathione peroxidase 1; CAT—catalase; Cu/Zn SOD—Cu/Zn superoxide dismutase. *p* < 0.05 low-Se vs. norm-Se group (*p* = 0.016).
